# A pediatric case of Burkitt’s lymphoma with bile duct obstruction requiring surgical biliary reconstruction

**DOI:** 10.1186/s40792-024-01935-5

**Published:** 2024-05-29

**Authors:** Shun Watanabe, Kan Suzuki, Kei Ogino, Sumiko Irie, Yuko Kamata, Shotaro Matsudera, Masahiro Hatanaka, Kazunori Kurosaki, Makoto Ishikawa, Mayuko Okuya, Yuya Sato, Akira Yamamiya, Atsushi Irisawa, Kazuyuki Ishida, Shigemi Yoshihara, Kazuyuki Kojima

**Affiliations:** 1 Department of Surgical Oncology, Graduate School of Medicine, Dokkyo Medical University, 880 Kitakobayashi, Mibu, Shimotsuga, Tochigi 321-0293 Japan; 2 Division of Pediatric Surgery, Dokkyo Children’s Medical Center, Dokkyo Medical University, 880 Kitakobayashi, Mibu, Shimotsuga, Tochigi 321-0293 Japan; 3 Department of Pediatrics, Dokkyo Medical University, 880 Kitakobayashi, Mibu, Shimotsuga, Tochigi 321-0293 Japan; 4 Department of Gastroenterology, Dokkyo Medical University, 880 Kitakobayashi, Mibu, Shimotsuga, Tochigi 321-0293 Japan; 5 Department of Diagnostic Pathology, Dokkyo Medical University, 880 Kitakobayashi, Mibu, Shimotsuga, Tochigi 321-0293 Japan

**Keywords:** Malignant lymphoma, Burkitt’s lymphoma, Biliary obstruction, Obstructive jaundice, Pediatric surgery, Biliary reconstruction, Choledochojejunostomy

## Abstract

**Background:**

Biliary obstruction due to compression by a B-cell solid tumor occurs rarely. A few reports have described biliary reconstruction surgery for obstructive jaundice caused by Burkitt’s lymphoma. However, there are no detailed reports on pediatric cases. We report a pediatric case of obstructive jaundice due to malignant lymphoma treated with biliary reconstruction surgery.

**Case presentation:**

A 5-year-old girl presented to our hospital with a massive abdominal tumor that caused biliary stricture. Chemotherapy was initiated after an open tumor biopsy. However, endoscopic biliary stent placement was performed owing to elevated bilirubin levels. We treated the patient with chemotherapy for 9 months while endoscopically replacing the biliary stent every few months. She achieved complete tumor remission. However, sclerotic lymph nodes were persistent on the dorsal side of the cholecystic duct junction, and biliary stricture at the same site had changed to stent-dependent biliary obstruction. Therefore, we performed choledochojejunostomy and retrocolic Roux-en-Y reconstruction 15 months after initial admission. There were no postoperative complications or tumor recurrences, and the bilirubin level remained low. Histopathologically, the resected bile duct wall was fibrotic and thick, and the bile duct lumen narrowed.

**Conclusions:**

Biliary reconstruction is effective to achieve long-term biliary patency in pediatric patients with stent-dependent biliary obstruction due to malignant lymphoma. However, the decision on when to stop biliary stent replacement and proceed to biliary reconstruction surgery is a matter of debate. Further case studies are required to address this issue.

## Background

Non-Hodgkin’s lymphoma (NHL) comprises only 1–2% of malignant biliary obstructions in adults [1]. Based on the clinical epidemiology of lymphoma, obstructive jaundice due to lymphoma occurs in approximately 1.3% of patients [2].

Thus, because obstructive jaundice caused by malignant lymphoma is rare and is often resolved with chemotherapy [3], there have been only a few reports of biliary reconstruction surgery in adults but none regarding children.

We report a pediatric case of obstructive jaundice due to malignant lymphoma treated with biliary reconstruction surgery.

## Case presentation

**Fig. 1:**
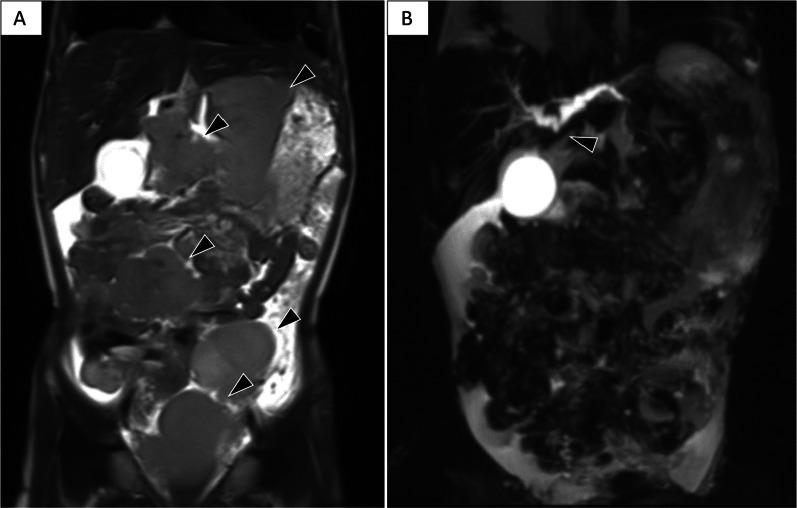
Magnetic resonance imaging at initial examination. **A** T2-weighted image shows multiple tumors occupying the abdominal cavity (arrowhead). **B** Maximum intensity projection image showing biliary obstruction at the level of the common hepatic duct (arrowhead)

A 5-year-old girl with no relevant medical history presented to our hospital with complaints of abdominal distention and pain, constipation, poor vitality, and jaundice. Computed tomography and magnetic resonance imaging (MRI) showed multiple masses extensively occupying the abdomen, common bile duct interruption, and dilation of the right and left hepatic ducts at the hepatic portal (Fig. 1).

On admission, her blood sample showed the following results: white blood cell count, 7.20 × 10^9^/L; hemoglobin, 11.9 g/dL; platelet count, 296 × 10^9^/L; aspartate aminotransferase, 286 U/L; alanine aminotransferase, 609 U/L; γ-glutamyl transferase, 301 U/L; total bilirubin, 5.3 mg/dL; direct bilirubin, 4.1 mg/dL; blood urea nitrogen, 11.3 mg/dL; creatinine, 0.51 mg/dL; total protein, 5.8 g/dL; and C-reactive protein, 1.3 mg/dL. The serum lactate dehydrogenase level was high (1237 U/L) on admission. No significant tumor markers were detected.

**Fig. 2:**
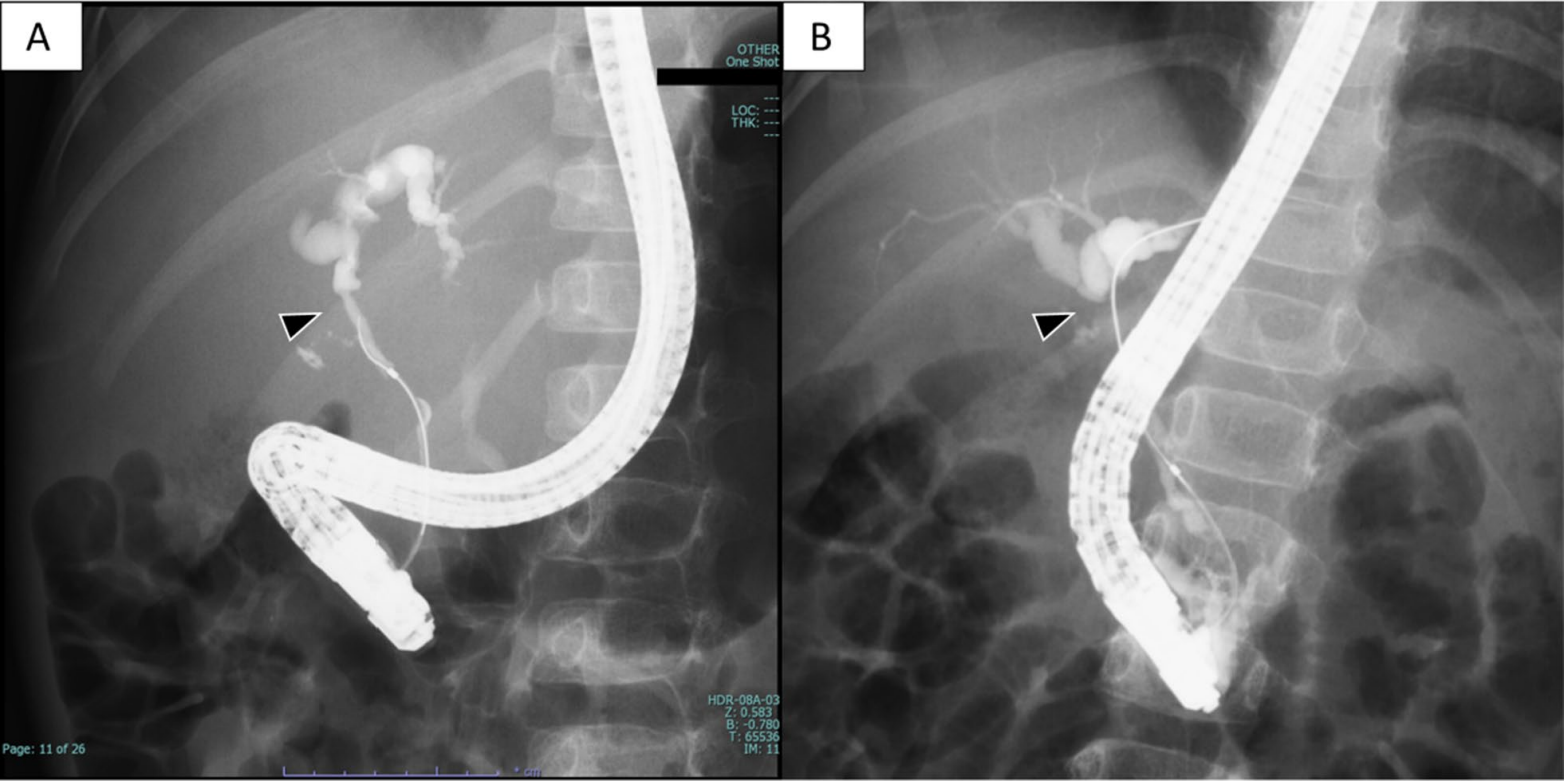
Endoscopic retrograde cholangiopancreatography before and after chemotherapy. **A** Initial biliary stricture (arrowhead). **B** Complete change in biliary obstruction at the junction of the cholecystic duct after chemotherapy (arrowhead)

The patient was hospitalized, and rapid tumor growth and pleural effusion were observed within a few days. Her serum lactate dehydrogenase level increased to 8787 U/L on the fifth day of admission. We performed a bone marrow biopsy, chest puncture, open tumor biopsy, and central venous catheter insertion on the same day. We initiated empirical chemotherapy promptly with cyclophosphamide and rituximab, and her bilirubin level decreased. The tumor was diagnosed histopathologically as Burkitt’s lymphoma. However, on the 14th day of hospitalization, the direct bilirubin level increased again to 8.0 mg/dL, and a plastic stent was placed endoscopically into the biliary tract. Endoscopic retrograde cholangiopancreatography (ERCP) at stent insertion showed biliary stricture (Fig. 2A).

Following biliary stent placement, the bilirubin level decreased. After that, we treated the patient with chemotherapy (two courses each of R-COPADM: rituximab, cyclophosphamide, vincristine, doxorubicin, and methotrexate; R-CYM: rituximab, cytarabine, and methotrexate; R-CYVE: rituximab, cytarabine, and etoposide). We administered chemotherapy for 9 months, during which time we replaced the stent endoscopically every 2–3 months.

After completing chemotherapy, thickening and stricture of the bile duct at the cholecystic duct junction persisted. A 7 mm lymph node without 18F-fluorodeoxyglucose accumulation was identified on the dorsal side of the common bile duct. However, it did not meet the criteria for residual disease with a maximum diameter of at least 10 mm [4]. Nevertheless, all target lesions were resolved, and we determined that the patient had achieved a clinical complete response. The patient developed biliary stent obstruction and cholangitis 2 months after chemotherapy. We performed an endoscopic biliary balloon dilation and a stent replacement. Although the jaundice improved temporarily, it did not lead to a fundamental improvement of the bile duct obstruction. ERCP during biliary stent replacement (Fig. 2B) and MRI showed progression from biliary stricture to biliary obstruction at the junction of the cholecystic duct. We determined that she had developed a stent-dependent biliary obstruction. However, we could predict that a choledochojejunostomy would be possible based on the location of the biliary obstruction in those images. The size of the lymph nodes on the dorsal side of the common bile duct remained unchanged for 9 months after the end of chemotherapy, and a clinical complete response was maintained.

**Fig. 3:**
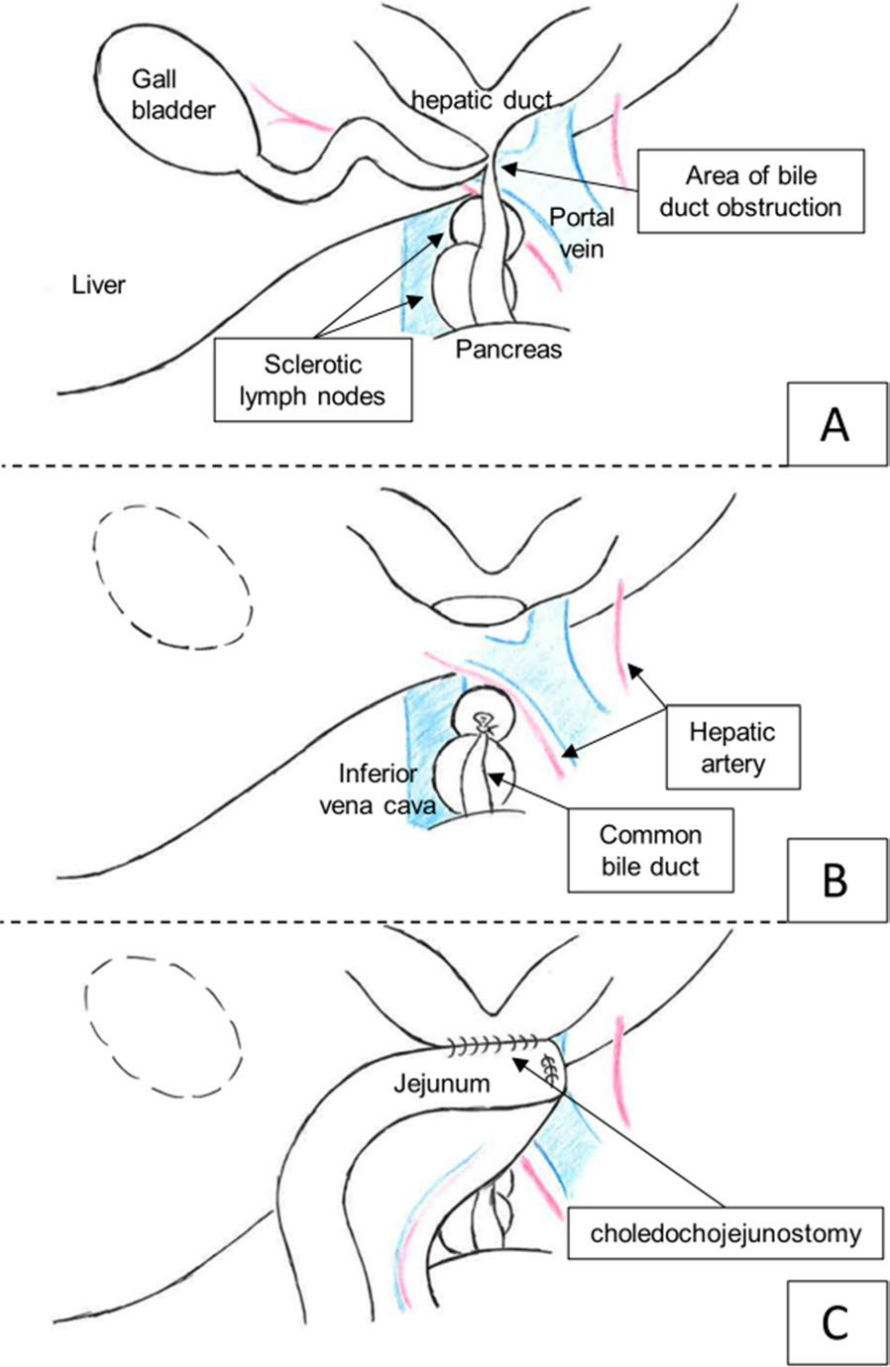
Intraoperative findings. **A** Area around the bile duct obstruction. **B** Schema after biliary resection. **C** Completed view after the choledochojejunostomy

**Fig. 4:**
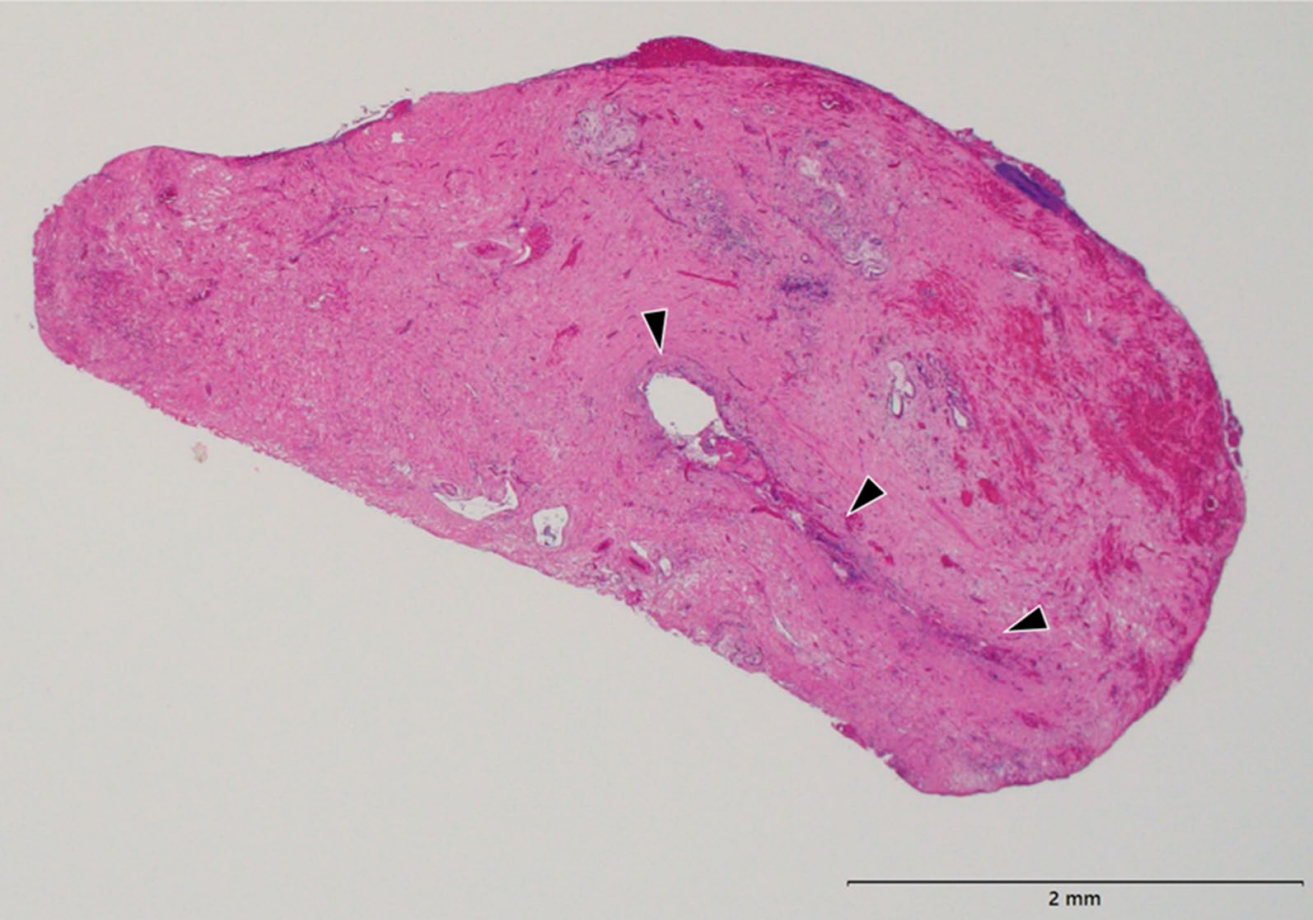
Histopathological findings of resected hepatic duct (hematoxylin–eosin stain, × 20). Fibrosis, thickening, and prolapse of the mucosa and muscularis propria at the wall of the resected hepatic duct. Narrowed bile duct cavity, approximately 2 mm (arrowhead)

**Fig. 5:**
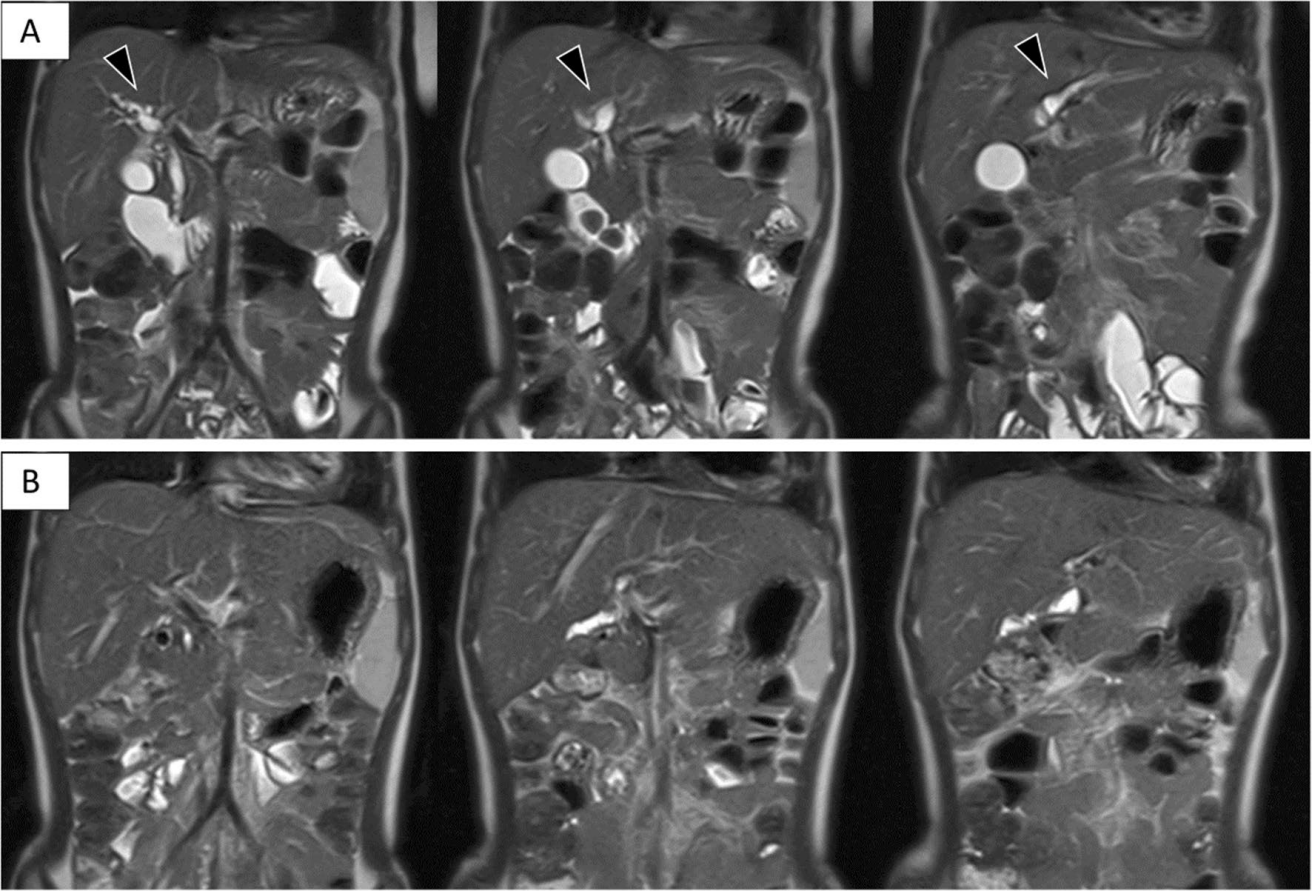
T2-weighted magnetic resonance imaging before and after choledochojejunostomy. **A** Intrahepatic bile duct dilatation before surgery (arrowhead). **B** Resolution of intrahepatic bile duct dilatation after surgery

We performed a biliary reconstruction procedure 15 months after her initial hospitalization (6 months after the end of chemotherapy). A 10 cm incision was made in the right hypochondrium. The bile duct wall was thickened and sclerosed, and indurated lymph nodes were observed on the dorsal side of the common bile duct. The adhesions were strong, but we dissected the dorsal portion from the gallbladder to the junction of the cholecystic duct. The cholecystic duct was cut at the junction, and the stent was removed. We resected the common hepatic duct and ligated the common bile duct. The common bile duct showed wall thickening and stricture, as observed in the preoperative images. In addition, sclerotic lymph nodes were observed between the common bile duct and portal vein. Choledochojejunostomy and retrocolic Roux-en-Y reconstruction were performed after resection of the hepatic duct up to the porta hepatis and bilateral incision of the hepatic duct to secure the anastomotic diameter (Fig. 3). The pace of growth of the Burkitt’s lymphoma remained rapid after 9 month post-chemotherapy. In addition, the portal vein was located near a lymph node on the dorsal portion of the common bile duct; thus, we chose to leave that node undisturbed to avoid the risk of injuring it. The intraoperative blood loss was 20 mL, and the operative time was 5 h and 20 min. Histopathologically, the resected bile duct wall showed fibrosis, thickening, and prolapse of the mucosa and muscularis propria. However, it revealed no malignant findings. The bile duct cavity had narrowed to approximately 2 mm (Fig. 4). There were no postoperative complications, and the patient remained in remission for 6 month post-surgery. MRI showed resolution of biliary congestion, and the bilirubin level remained low (Fig. 5).

## Discussion

Lymphoma leading to jaundice is rare, and there is no constant method for its management [5]. Most NHL cases leading to jaundice are diffuse B-cell lymphomas. The causes of jaundice include idiopathic and tumor-related hemolysis, hepatic invasion of the tumor, and biliary obstruction due to tumor compression. Biliary obstruction due to tumor compression is considered the most common cause [6]. Benign obstruction is the compression of the bile ducts by tumors or lymph nodes and radiation-induced biliary obstruction, as opposed to development from the bile ducts or malignant invasion into the bile duct. Resected specimens of benign biliary obstruction in malignant lymphoma cases that were not treated with radiation therapy show primary sclerosing cholangitis-like fibro-adipose degeneration of the biliary wall pathologically [7]. Pathological examination of the resected bile duct in the present case showed fibrosis, thickening, and prolapse of the mucosa and muscularis propria. The bile duct obstruction in the present case could be attributed to malignant lymphoma invasion into the bile duct, wall thickening due to compression by the tumor, inflammation due to cholangitis, or scarring due to anticancer drugs. However, determining the cause is difficult.

The bile duct can be obstructed by lymphoma at any site; the most common sites are the porta hepatis and peripancreatic area. This is assumed because the bile ducts are relatively fixed and not mobile in these areas [1, 6]. Long-term follow-up is essential because biliary obstruction due to malignant lymphoma may occur initially but often occurs during treatment or after tumor remission [7]. In the present case, the patient had a biliary stricture near the junction of the cholecystic duct since the first visit.

Burkitt’s lymphoma is a B-cell solid tumor that accounts for approximately 40% of pediatric NHL [8]. Chemotherapy is the primary treatment for pediatric patients with abdominal Burkitt’s lymphoma. Surgery should be limited to biopsy if complete resection is not possible because volume reduction delays the start of chemotherapy and does not improve prognosis [3]. Therefore, surgeons are rarely involved in the treatment of Burkitt’s lymphoma.

In the management of Burkitt’s lymphoma with biliary obstruction, 20 of 47 patients (42.6%) had resolution of obstruction with only primary chemotherapy, which is expected to be relieved even without biliary drainage [7]. By contrast, some recommend chemotherapy after biliary drainage because biliary obstruction may increase the risk of using chemotherapeutic agents with high hepatic elimination, such as doxorubicin and vinblastine [5, 7, 9]. In the present case, although we attempted prior chemotherapy, the worsening biliary stricture required stent insertion. Regarding endoscopic biliary stenting, the success rate of insertion is not low, with 26 of 35 patients (74.3%) reported to have undergone stenting transendoscopically [5]. Regarding removal of the inserted stent, the combined results of the two previous reports show that biliary stents could be removed in 22 of 52 patients (42.3%) after chemotherapy [5, 7]. Based on these data, jaundice cannot be controlled with chemotherapy alone in 57.4% [(1–0.426) × 100]. Furthermore, stenting is impossible in 25.7% [(1–0.743) × 100], and stent removal is impossible after chemotherapy in 42.9% [0.743 × (1–0.423) × 100]. Therefore, the failure rate of combining chemotherapy and stenting to control jaundice is approximately 40% (39.4%: 0.574 × (0.257 + 0.429) × 100), which is not rare.

In addition, when transendoscopic plastic stent replacement, usually performed approximately every 3 months, is no longer possible [5] or when biliary stents do not drain effectively [8], open biliary reconstruction must be performed for reliable early drainage. When stent-dependent bile duct obstruction persists after chemotherapy, as in the present case, the timing of stent replacement or biliary reconstruction surgery, even in adults, must be determined on a case-by-case basis. In the present case, we performed biliary reconstruction because the patient had stent obstruction and cholangitis and we could predict that choledochojejunostomy was feasible. Our decision to perform biliary reconstruction instead of continued stent replacement was correct based on the operative and pathological findings of severe bile duct stricture. Publication bias should undoubtedly be considered. However, the study by Odemiş et al. [7] did not report complications of surgical biliary reconstruction for biliary obstruction due to lymphoma in the 26 adult patients studied. In our experience, this procedure is difficult because it is performed at the site of previous inflammation caused by the tumor.

In adult patients with biliary cancer, metal stents are sometimes used for biliary drainage instead of continuously replacing the plastic stents [5]. In children, the use of metallic stents is not recommended because they are difficult to be replaced when stent occlusion occurs or when growth creates the need to increase the stent size because of the possibility of secondary biliary obstruction due to granulation caused by prolonged implantation of rigid stents. Our experience suggests that surgical biliary reconstruction is an effective treatment for long-term biliary patency in children.

## Conclusions

Biliary reconstruction is effective for long-term biliary patency in pediatric patients with malignant lymphoma and stent-dependent biliary obstruction. However, the decision on when to stop biliary stent replacement and proceed to biliary reconstruction surgery is a matter of debate. Future case studies are needed to elucidate this further.

## Data Availability

Data sharing is not applicable to this article as no new data were created or analyzed in this study.

## Abbreviations

NHL: Non-Hodgkin's lymphoma
MRI: Magnetic resonance imaging
ERCP: Endoscopic retrograde cholangiopancreatography
